# A New Israeli *Tobamovirus* Isolate Infects Tomato Plants Harboring *Tm-2^2^* Resistance Genes

**DOI:** 10.1371/journal.pone.0170429

**Published:** 2017-01-20

**Authors:** Neta Luria, Elisheva Smith, Victoria Reingold, Ilana Bekelman, Moshe Lapidot, Ilan Levin, Nadav Elad, Yehudit Tam, Noa Sela, Ahmad Abu-Ras, Nadav Ezra, Ami Haberman, Liron Yitzhak, Oded Lachman, Aviv Dombrovsky

**Affiliations:** 1 Department of Plant Pathology, ARO, The Volcani Center, Rishon LeZion, Israel; 2 Department of Vegetables and field crops, ARO, The Volcani Center, Rishon LeZion, Israel; 3 Electron Microscopy Unit, Departments of Chemical Research Support, Weizmann Institute of Science, Rehovot, Israel; 4 Plant protection and inspection services, Beit-Dagan, Israel; 5 Department of Plant Sciences, George S. Wise Faculty of Life Sciences, Tel Aviv University, Tel Aviv, Israel; Oklahoma State University, UNITED STATES

## Abstract

An outbreak of a new disease infecting tomatoes occurred in October-November 2014 at the Ohad village in Southern Israel. Symptomatic plants showed a mosaic pattern on leaves accompanied occasionally by narrowing of leaves and yellow spotted fruit. The disease spread mechanically and rapidly reminiscent of tobamovirus infection. Epidemiological studies showed the spread of the disease in various growing areas, in the South and towards the Southeast and Northern parts of the country within a year. Transmission electron microscope (TEM) analysis showed a single rod-like form characteristic to the *Tobamovirus* genus. We confirmed Koch’s postulates for the disease followed by partial host range determination and revealed that tomato cultivars certified to harbor the *Tm-2*^*2*^ resistance gene are susceptible to the new viral disease. We further characterized the viral source of the disease using a range of antisera for serological detection and analyzed various virus genera and families for cross-reactivity with the virus. In addition, next generation sequencing of total small RNA was performed on two cultivars grown in two different locations. In samples collected from commercial cultivars across Israel, we found a single virus that caused the disease. The complete genome sequence of the new Israeli tobamovirus showed high sequence identity to the Jordanian isolate of tomato brown rugose fruit virus.

## Introduction

Tomato plants (*Solanum lycopersicum*) with edible fruit are important vegetables in the world diet [1, 2]. Trellised tomato plants grown in protected structures, greenhouses, net-houses are highly exposed to infections by mechanically transmitted viruses or viroids primarily by the prevalent genera of the *Tobamoviruses*, *Potexviruses* and *Pospiviroids*. According to the 2015 release of the International Committee on Taxonomy of Viruses (ICTV) http://www.ictvonline.org/virustaxonomy.asp, the *Tobamovirus* genus is the largest genus (35 species) among the seven genera in the family *Virgaviridae*. The *Tobamovirus* genus includes the well-known species the type member *Tobacco mosaic virus* (TMV) [3] and the *Tomato mosaic virus* (ToMV) as well as *Tobacco mild green mosaic virus* (TMGMV) and *Pepper mild mottle virus* (PMMoV) among the viruses capable of infecting *Solanaceae* crops [4, 5]. *Tobamoviruses* are characterized by a typical rod-shaped particle morphology encapsulating a single stranded RNA (+ssRNA) sense genome of 6.2 to 6.4kb encoding four ORFs. ORF1 and ORF2 are separated by a leaky stop codon and encode non-structural proteins that form the replicase complex. ORF3 on the large subgenomic RNA encodes the non-structural movement protein (MP). ORF4 on the small subgenomic RNA encodes the coat protein (CP) of 17 to 18 kDa. Tobamoviruses are transmitted by mechanical contact: through workers' hands, clothes, tools, and are capable to preserve infectivity in seeds and contaminated soil [6, 7]. In tomatoes, dominant resistance introduced by introgression resulted in resistance to TMV and ToMV by the R genes *Tm-2* and *Tm-2*^2^ (*Tm-2*^*a*^) respectively [8–12]. The *Tm-2* and *Tm-2*^*2*^ resistances share the viral MP as the *Avirulence* protein (Avr). However, different domains in the MP and different protein structure requirements are necessary for each resistance [10, 13–15]. The *Tm-2*^*2*^ resistance has been more durable than the *Tm-2*, which has been broken [10, 16, 17]. However, concern for the effectiveness of *Tm-2*^*2*^ resistance rises since new tobamoviruses infecting tomatoes were identified. In Mexico, a tobamovirus named *Tomato mottle mosaic virus* (ToMMV) [18] and in Jordan a tobamovirus putatively named tomato brown rugose fruit virus (TBRFV-Jo)[19]. ToMMV causes tissue necrosis of the leaves of tomato seedlings and mosaic and leaf distortion of mature plants. TBRFV-Jo causes mild foliar symptoms but brown rugose symptoms on fruits. Here we describe an outbreak of a disease, which occurred in October to November 2014 in tomato crops of cultivars (cvs.) Mose and Ikram in Israel non-grafted or grafted on rootstock cv. Arnold, grown in six 50-mesh net houses in 30 acres in Ohad village in the South of the country. The disease symptoms include mild and severe mosaic on leaves with occasional leaf narrowing. Yellow spotted fruits estimated to amount to 10 to 15% of the total fruit were detected on each symptomatic plant. The goal of the present study was first to characterize the disease-causing agent and to identify the potential host range of the disease for risk assessment. The second goal was to obtain the complete genomic sequence to determine the species affiliation of the new virus in the *Tobamovirus* genus.

## Materials and Methods

### Virus purification and transmission electron microscope (TEM) analysis

Symptomatic tomato fruit and leaves were collected from infected symptomatic plants. Virions were purified from 100 g of symptomatic plants, as described previously [20]. Leaf-dip analysis was carried out with 0.2g symptomatic tomato leaves that were ground in 0.01 M phosphate buffer pH 7.0. After centrifugation in 10,000 g for 15 min the supernatant was analyzed in TEM. For TEM analysis, 3.5μL of sample was applied to glow-discharged, homemade 300 mesh carbon-coated copper TEM grids for 30 seconds. Excess liquid was blotted and after a wash with distilled water, the grids were stained with 2% uranyl acetate. Samples were visualized in an FEI Tecnai T12, TEM operated at 120 kV, equipped with a Gatan ES500W Erlangshen camera. 242 viral particles from two separate viral preparations were measured in TEM images. Scaling was done using a standard of known size measured at different magnifications. Viral length of each particle was measured by stretching a line from end to end.

### Establishment of continuous virus cultures and host range analysis

Purified viral particles served for mechanical inoculation of laboratory test plants and tomato plants harboring the *Tm-2*^*2*^ resistance gene. Symptomatic tomato plants from the initial experiments served for sap-mechanical inoculation assays. Tomato fruit and leaves were ground in 0.01 M phosphate buffer (pH 7.0) and inoculated to a variety of potential host plants (pre-dusted with carborundum) for extended host range determination. Host range testing was performed in three sets of experiments (> four plants per species). Inoculation of a range of commercial tomato varieties harboring the *Tm-2*^*2*^ resistance gene, as certified by the 'Tomato Genetic Resource Center' (TGRC) http://tgrc.ucdavis.edu/Data/Acc/dataframe.aspx?start=AccSearch.aspx&navstart=nav.html, was performed with 8 replicates.

The virus was maintained on systemically infected tomato plants (harboring the *Tm-2*^*2*^ resistance gene) cv’s: Ikram, Odelia, Bruno, Olympiacos, Magi, Tory which served as propagation hosts. In addition to the registered resistance to ToMV by the seed-companies, the authenticity of the presence of *Tm-2*^*2*^ was further confirmed by DNA sequencing of the resistance gene in Odelia and Ikram cultivars. All virus-infected plants were kept in an insect-proof growth chamber inside a glasshouse and sprayed regularly with insecticides to prevent any infestation.

### Protein separation on sodium dodecyl sulfate polyacrylamide gel electrophoresis (SDS-PAGE), Coomassie staining and Western blot analysis

Purified viral particles and plant extracts were mixed with loading buffer [21] and separated on SDS-PAGE. Proteins were stained with Coomassie brilliant blue G-250 (Sigma-Aldrich) for 1 hour followed by overnight de-staining with 10% glacial acetic acid and 20% methanol. Western blot assay was carried out with the various preparations as previously described [22]. Total plant proteins were extracted from four leaf discs harvested from infected and uninfected plants. Each sample was ground in 0.5 ml urea-SDS-ß-mercaptoethanol (USB) buffer (75mM Tris-HCL (pH6.8), 9 M urea, 4.5% (g/v) SDS and 7.5% (v/v) ß-mercaptoethanol). The extracted samples were boiled for 10 min at 95°C and then cooled on ice for 5 min followed by 10 min centrifugation at 13,000 g. Then 100μl aliquots of the supernatant were mixed with 35 μl 4x loading buffer. Samples were separated on 15% SDS-PAGE. The gels were electro-blotted onto nitrocellulose membrane for 40 min at 245 mA for 2 membranes using semidry transfer apparatus (Bio-Rad). The membranes were blocked with PBS containing 3% nonfat milk powder for 2 h at room temperature and then the antisera was added at different dilutions, for overnight incubation at 4°C. Then three washes with PBS-Tween were applied. Following that, commercial secondary anti-rabbit antibodies with alkaline phosphatase conjugate (Sigma) were added (1:5,000) and incubated for 1 h at room temperature with slow agitation. Following three washes with 5 min intervals, the proteins were visualized by adding the substrate (NBT, BCIP; Promega).

### Antisera preparation

Purified viral particles were subjected to protein separation on SDS-PAGE to determine the quality and purity of the samples and were then served for antisera preparation (Adar Biotech Rehovot, Israel). Pre-immune blood was collected from two New Zealand white rabbits three months old. Native purified virus preparation (with no SDS or boiling) was diluted with saline, combined with complete Freund’s adjuvant was injected subcutaneously to the rabbits to create enhanced immune response. Three sequential injections followed, at 21 day intervals in which the antigen was mixed with incomplete Freund’s adjuvant. Ten to thirteen days following the fourth injection the rabbits were bled and sera (40 ml) was separated from blood cells and served for evaluation by direct ELISA and Western blot analysis prior to IgG purification.

### Enzyme-linked immunosorbent assay (ELISA)

Direct and Double Antibody Sandwich (DAS) ELISA were performed on various plant preparations as previously described [23] using laboratory-produced antisera or antibodies for TMV, ToMV and the antisera raised against the purified virus preparation of the new tobamovirus isolate of the current study.

The diagnosis of *Tomato spotted wilt virus* (TSWV) was performed using TSWV specific antibodies in a commercial kit (Agdia). The optical density (O.D) readings of hydrolyzed substrate of Alkaline-Phosphatase (Sigma) were measured at 405 nm.

### Extraction and characterization of viral RNA

For RNA extraction, purified viral preparations served as the source material as described previously [24] with modifications [25]. Briefly, virions were incubated with RQ RNase-free DNase I (Promega) for 1 h at 37°C, followed by proteinase K (Sigma) treatment at a final concentration of 200 μg/ml for 1 h at 37°C. Viral nucleic acids were further purified and precipitated with acidic phenol (Ambion/Applied Biosystems). The aqueous phase was precipitated overnight at -20°C in the presence of glycogen (Fermentas), 0.1 M sodium acetate and 3 to 4 volumes of isopropanol. The precipitated viral RNA was washed with 70% ethanol and allowed to air dry for 10 min. The dry viral RNA was suspended in double distilled water.

### Reverse transcription (RT) and PCR amplification

For the RT reaction viral RNA served as a template using the Maxima Reverse Transcriptase cDNA kit (Thermo Fisher Scientific). General tobamovirus primer sets were designed according to consensus sequences that we identified from complete genome sequences of tomato-infecting tobamoviruses from the GenBank. The R-4718: 5’-CAATCCTTGATGTGTTTAGCAC-3’ reverse complement primer was used for the RT reaction. The resulting cDNA was amplified by PCR using Taq DNA polymerase JMR PCR mix (JMR Holdings) and the designed general primer set F-3666: 5’-ATGGTACGAACGGCGGCAG-3’ and R-4718. Additional primer sets that were used for genome amplification for validation of the NGS obtained contigs are described in S1 Table. For TSWV diagnosis, viral RNA served as a template for cDNA synthesis using the reverse complement primer R-Tswv-NC-2770 5’…GATCATGTCTAAGGTTAAGC…3’ followed by PCR amplification with the supplement of the forward primer F-Tswv-NC-1980 5’…CAGCTGCTTTCAAGCAAGTTC…3’. The resulting amplicons were cloned into pGEM-T-easy vector and sequenced in both orientations. Sequence homology was determined using BLAST (http://blast.ncbi.nlm.nih.gov/Blast.cgi) algorithms.

### Next generation sequencing (NGS) with Illumina MiSeq and phylogenetic analysis

For NGS analysis, two different symptomatic tomato plants samples positively detected by ELISA, western blots and RT-PCR were selected. The first sample of cv. Mose exhibited the typical disease symptoms, was collected in October 2014 from Ohad village in Southern Israel. The second sample of cv. Odelia, which exhibited distinct disease symptoms, was collected in May 2016 from Sde-Nitzan village also in Southern Israel. Symptomatic samples were subjected to total RNA extraction using TRI reagent (Sigma-Aldrich), followed by mirVana miR Isolation kit (Invitrogen) for enrichment of small RNA molecules. The obtained small RNA fraction was used to construct a small RNA library using the TruSeq Small RNA Sample Preparation kit (Illumina). Library fragment size and quality of sample purification were analyzed using the Agilent 2100 Bioanalyzer (Agilent Technologies). Size selection of the complete library was performed using E-Gel EX 4% agarose gels (Invitrogen/Life Technologies). The library was sequenced using the MiSeq Sequencer (Volcani Center, Israel). Phylogenetic tree analysis was carried out for concatenated ORF amino acid sequences. First, the MAFFT program was used to align nucleotides or ORF [26]. Then, a phylogenetic tree was constructed based on a maximum-likelihood framework, using the PhyML3.0 software with 1,000 bootstrap replicates [27].

### Genome assembly and bioinformatics sequence analysis

The 3' adapter sequence was removed and sequences were filtered for size selection. The size range selected was between 19 and 30 bp in length using the software UEA sRNA Workbench version 2.5 [28]. After sequence cleaning and filtering, the short reads were assembled using the Velvet assembler [29] with multiple k-mer lengths from 9 to 23. The different assemblies were merged using AssemblyAssembler1.3 Python script (https://github.com/dzerbino/velvet/tree/master/contrib/AssemblyAssembler1.3). Generated contigs were then BLASTed using blastx algorithm against the non-redundant NCBI protein database (NCBI, 2014).

### Obtaining the sequences of the 5’ and 3’ untranslated regions (UTR)

The 5’- and 3’-UTR sequences of the viral genome were obtained using a RACE kit (Invitrogen) according to the manufacturer’s protocol, on a template of viral RNA extracted from purified virus preparations. The RACE procedure was repeated three times on different virus preparations to ascertain the accuracy of the UTRs.

### Survey analysis

The Israeli "Plant Protection and Inspection Services” (PPIS) survey was conducted in February 2015. Sampling for disease detection was executed on a third of each farmer's growing area up to three structures per grower. From each structure, thirty sub-samples were collected and divided into three pools of ten samples each for ELISA. Selected ELISA-negative and all the ELISA-positive samples were tested by RT-PCR amplification followed by amplicon sequencing for validation. In addition, for selected samples, negative and positive ELISA and RT-PCR analysis were further tested by inoculation of susceptible *Nicotiana tabacum* cultivars (bioassay) (S1 Fig).

## Results

### A new tobamovirus isolate infects tomato plants in Israel

**Fig 1:**
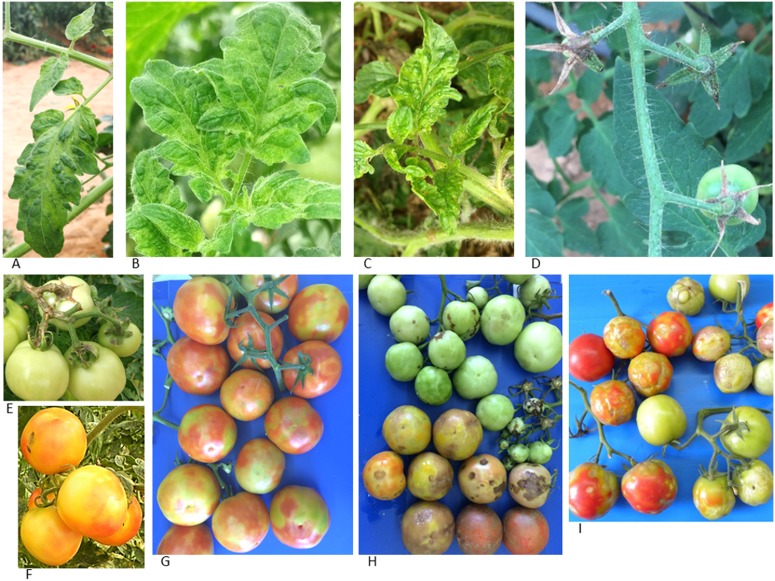
Naturally infected tomato plants. **(A-C)** Symptomatic mosaic pattern on leaves of cluster tomato plants cv. Mose. **(C)** Narrowing leaves of cluster tomato plants. **(D)** Dried peduncles and calyces on cherry tomato plants cv. Shiran leading to fruit abscission. **(E)** Necrotic symptoms on pedicle, calyces and petioles cv. Ikram. **(F)** Typical fruit symptoms with yellow spots cv. Mose. **(G-I)** Variable symptoms of tomato fruits cv. Odelia. **(G)** The typical disease symptoms. **(H)** Symptoms of mixed infections by the abundant TSWV and the new tobamovirus isolate. (**I**) Unique symptoms of the new tobamovirus isolate found at a single location at Sde-Nitzan village.

**Fig 2:**
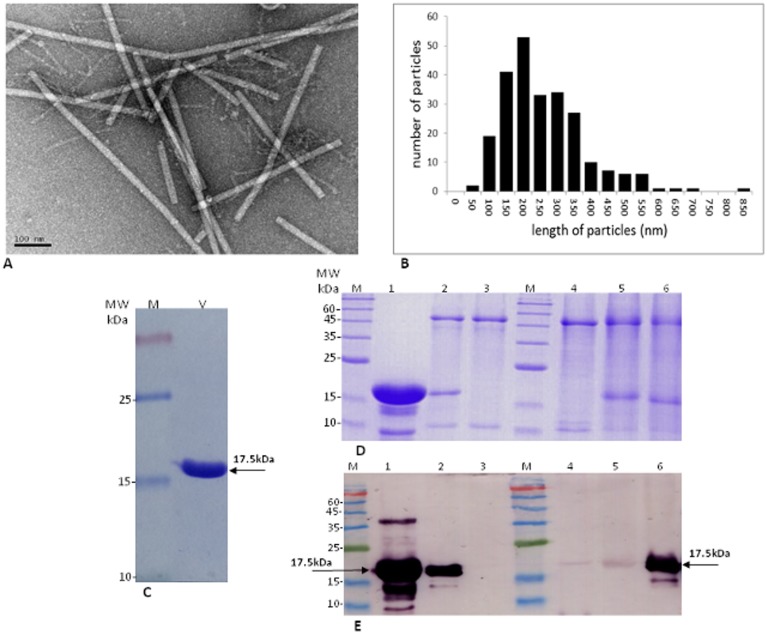
Morphological and serological characterization of viral particles and coat protein. **(A)** Electron micrograph illustration of viral particles. (B) Distribution of viral particle lengths as imaged by TEM showing average size of 235±123nm. (**C**) SDS-PAGE (15%) analysis of viral particles preparation followed by Coomassie brilliant blue staining depicting the CP at molecular mass of ~17.5 kDa. (D-**E**) Testing the specificity and the cross reactivity of the antisera raised against the new tobamovirus isolate. By Coomassie brilliant blue staining (D) in parallel to Western blot analysis (E). Purified virions (1), CP from infected tomato plants extract, cv. Ikram (2). An extract from healthy (non-infected) tomato leaves (3). Cross reactivity of the antisera with (4) extracted pepper leaves, cv. Maor infected with PMMoV, (5) extracted *Nicotiana tabacum* cv. *Samsun* infected with TMGMV and (6) extracted tobacco leaves cv. Samsun infected with TMV.

Naturally infected tomato plants cv. Mose or Ikram non-grafted or grafted on 'Arnold' rootstock were discovered in October 2014 in six net houses (30 acres total) of a single farm at Ohad village in Southern Israel. The disease symptoms were mild and severe mosaic of the leaves with occasional narrowing of the leaves. Yellow spotted fruit that amounted to 10 to 15% of the total fruit occurred on symptomatic plants (Fig 1 and S2 Fig). The naturally infected tomato plants were collected for laboratory analysis to identify the disease-causing agent. Viral particles purified from symptomatic plants were visualized by TEM. Single viral particle morphology with a rigid rod-like form resembling the *Tobamovirus* genus was observed (Fig 2A). In viral preparation, the purified viral particles as imaged by TEM appear to have an average size of 235±123nm in length (Fig 2B) and 18nm in width. Similarly, in leaf-dip analysis, while most of the particles were ~300 nm long, particles of ~500 nm long and small particles of ~250 nm long were observed as well (S3 Fig). SDS-PAGE protein separation of purified virus preparation followed by Coomassie staining allowed the visualization of a single dominant CP of ~17.5 kDa (Fig 2C).

### Host range determination

**Fig 3:**
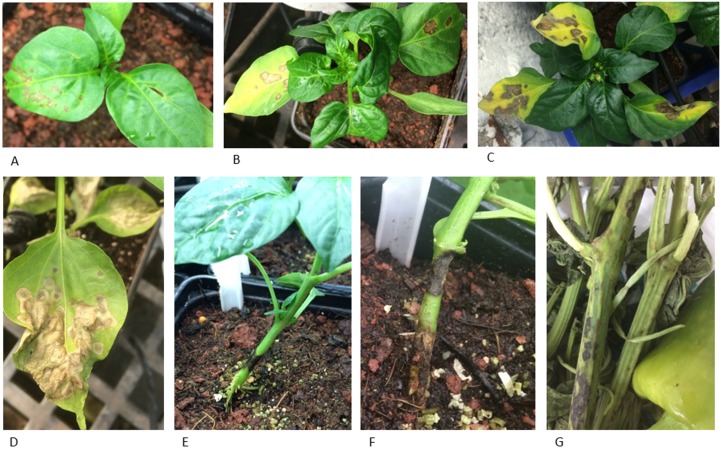
Pepper plants harboring *L*^*1*,*3*,*4*^ hypersensitivity response (HR) to infection by the new tobamovirus isolate. **(A-D)** Symptoms developed following sap-mechanical leaves inoculation showing **(A)** necrotic lesions; **(B)** yellowing; **(C, D)** dried apoptotic leaves. **(E-G)** HR symptoms developed following root inoculation demonstrating dried spots on stems leading to plant growth inhibition.

Partial host range analysis of the new Israeli tobamovirus isolate (the details of virus identification are below) was carried out in two steps. At first, viral particles purified from the source tomato plants were inoculated to healthy tomato plants cv. Ikram and tobacco plants. Secondly, the inoculated tomato plants served for sap-mechanical inoculation of additional commercial tomato varieties carrying the *Tm-2*^*2*^ resistance (Table 1A and 1B) and of laboratory test plants (Table 2). In all the inoculated tomato cultivars that we tested (of the cluster type), systemic symptoms developed at 12–18 days post inoculation (dpi). Unlike ToMV, the new tobamovirus isolate caused systemic infection of all tomato cultivars tested harboring the *Tm-2*^*2*^ resistance as certified by the Tomato Genetic Resource Center (TGRC) (Table 1A and 1B). Variations in susceptibility to viral infection were observed among the *Solanaceae* family. In pepper, *Capsicum annuum* genotypes harboring the *L*^*1*,*3*.*4*^ resistance genes, hypersensitivity response (HR) developed on the inoculated leaves at 4 dpi, and then at 7 dpi leaf yellowing symptoms occurred, followed by leaf fall at 9–12 dpi (Fig 3A–3D and S4 Fig). When pepper plants are root inoculated and propagated at warm temperatures (above 30°C), the HR response includes necrotic lesions on the roots and stem that inhibit plant growth and often leads to plant collapse (Fig 3E and 3F). In petunia hybrids, potatoes and eggplants no visual symptoms were observed 21–30 dpi (Table 2). Local and necrotic lesions developed in the inoculated tobacco species *N*. *benthamiana*, *N*. *glutinosa*, and *N*. *sylvestris* 4–7 dpi and collapsed plants were observed 7–14 dpi. Importantly, the virus infects common weeds as well (Table 2). The *S*. *nigrum* (black nightshade) and *Chenopodium murale* were identified as potential reservoirs for the virus. *S*. *nigrum* was asymptomatic for virus infection while *C*. *murale* shows a hypersensitive response (HR) by necrotic leaf yellowing followed by local necrotic lesions prior to symptomless systemic infection.

**Table 1:** A new tobamovirus isolate infects commercial tomato cultivars harboring *Tm-2*^*2*^ resistance gene.

Accession number	Genotype specification	DSI (av)	ELISA (av)
LA2706 (Moneymaker)	Susceptible	1.6	0.6473
LA2399	*S*. *lycopersicum* cv. T-5 (*Tm-2*)	0.3	0.0108
LA3027	*S*. *lycopersicum* cv. Vendor (*Tm-2*)	0	0.7306
LA2828	*S*. *lycopersicum* cv. Momor (*Tm-2*^*2*^)	0	0.005
LA2830	*S*. *lycopersicum* cv. Mocimor (*Tm*, *Tm-2*^*2*^)	0.4	0.0053
LA2968	*S*. *lycopersicum* cv. Vendor (*Tm-2*^*2*^)	0	0.0050
LA3310	*S*. *lycopersicum* cv. Moneymaker (*Tm-2*^*2*^)	0	0.0

LA2706 (Moneymaker)	Susceptible	2.7	0.802
LA2706 (Moneymaker)	Susceptible	2.9	0.865
LA2827	*S*. *lycopersicum* cv. Moperou (*Tm-2*^*2*^)	3	0.829
LA2829	*S*. *lycopersicum* cv. Momor verte (*Tm-2*^*2*^)	2.6	0.396
LA2830	*S*. *lycopersicum* cv. Mocimor (*Tm*-1, *Tm-2*^*2*^)	2.4	0.659
LA2399	*S*. *lycopersicum* cv. T-5 (*Tm-2*)	2.8	0.647
LA2828	*S*. *lycopersicum* cv. Momor (*Tm-2*^*2*^)	3	0.889
LA2968	*S*. *lycopersicum* cv. Vendor (*Tm-2*^*2*^)	2.9	0.733
LA3310	*S*. *lycopersicum* cv. Moneymaker (*Tm-2*^*2*^)	3	1.13

**A.** Mechanical inoculation of *Tomato mosaic virus* (ToMV). **B**. Mechanical inoculation of the new tobamovirus isolate, current study. (**DSI**) Disease symptom Severity Index. (**av**) Average of 7–8 samples. **DSI ratings**: 0-no symptoms; 1-mild mosaic; 2-severe mosaic; 3-narrowing leaves. Accession number as registered in the Tomato Genetic Resource Center (TGRC) database, http://tgrc.ucdavis.edu/.

**Table 2:** Partial host range analysis following sap-mechanical inoculation of the new tobamovirus isolate.

Host plant	Early symptoms 4–7 dpi	Systemic symptoms description 7–14 dpi	ELISA
***Cappsicum annumm* (pepper)** *Cv’s Maor, (*L*^***1***^**), Fiona (*L***^**3,**^ ***Sw-5*), Romans and Lyri (*L***^***4***^**, *Sw-5*)**	HR	NS	+
*Chenopodium murale*	YNL, BNL	MM, LL	+
*C*. *amaranticolor*	YNL	NS	+
*C*. *quinoa*	YNL	NS	+
*Datura stramonium*	YNL	NS	-
*Nicotiana benthamiana*	NL	LL, PC	+
*N*. *clevelandii*	BNL	LY	+
*N*. *glutinosa*	NL	MM	+
*N*. *tabacum cv*. *occidentalis*	M	MM	+
*N*. *tabacum cv*. *rustica*	+	MM	+
*N*. *tabacum cv*. *samsun*	MM	M	+
*N*. *tabacum cv*. *samsun N*.*N*	NL	NS	-
*N*. *tabacum cv*. *sylvestris*	NL	MM	+
*Petunia hybrida*	-	NS	+
*Solanum tuberosum* (potato) cv. Nicola	-	NS	-
*S*. *nigrum* (black nightshade)	-	MM/NS	+
*S*. *melongena* (eggplant) cv’s. Classic, 206	-	NS	-

* resistance genes: *L*, for tobamovirus, *Sw-5* for *Tomato spotted wilt virus* (TSWV). Early symptoms, local symptoms developed on the inoculated leaf 4–7 days post inoculation (dpi): hypersensitivity response **(HR)**, necrotic lesions **(NL)** yellowing necrotic lesions **(YNL)**, brown necrotic lesions **(BNL)**, mottling **(M)**. Systemic symptoms description 7–14 days post infection: no symptoms (NS); mild mottling (MM); Leaf lesion (LL). Mosaic (M); plant collapse (PC); Leaf yellowing (LY). Positive ELISA results, >3 of negative value are depicted in (+), while negative results marked (-).

At 21 dpi newly emerged leaf samples were collected from inoculated host plants and subjected to ELISA for virus detection. The susceptibility of the hosts to the virus inoculation was analyzed again at 30 dpi (Table 2). Laboratory produced antibodies for anti-TMV and anti-ToMV that were used for diagnosis showed limited specificity to the new tobamovirus isolate in ELISA. Occasionally, symptomatic plants were not detected by the antisera (values were lower than 3 times of the negative control, data not shown). However, antisera raised against the purified viral preparation (Fig 2C) of the new Israeli tobamovirus isolate showed high specificity for the purified viral CP (Fig 2E).

In fulfillment of Koch's Postulates, the results based on symptom development and ELISA clearly showed that all the tomato varieties harboring the *Tm-2*^*2*^ resistance were susceptible to virus inoculation (Table 1A and 1B). Symptom development was similar to those of the original source plants (Fig 1). Virus purification from the secondarily infected tomato plants showed typical tobamovirus particle morphology in TEM, although wide size range of viral particles was observed (Fig 2B). Further characterization of the disease-causing agent by serological assay (ELISA, Western blot), amplicon sequencing and NGS are described below.

### Serological characterization of the new infectious tobamovirus isolate

Purified particles of the new infectious tobamovirus isolated from infected plants served to raise antibodies against the virus CP as mentioned above. The antiserum was used for serologically-based identification, by ELISA (dilution 1:12,000) and Western blot analyses (dilution 1:10,000). In ELISA the average O.D readings following 20 minutes of substrate hydrolysis (color development) at room temperature were 0.7 ±0.2 O.D which is >50 times the negative control samples that were 0.015 ± 0.02 O.D. In Western blot, the antiserum allowed specific identification of a ~17.5 kDa viral CP in extracts of infected plants, characteristic of tobamoviruses (Fig 2E). Analysis of antibody specificity to the purified virus showed cross reactivity with the CP of TMV and PMMoV. A slight cross reactivity was observed with the CP of TMGMV (Fig 2E), although all these viruses belong to the *Tobamovirus* genus that infect the *Solanaceae*.

### NGS and genome assembly

Small RNA was extracted from symptomatic leaf samples of two different tomato cultivars collected from two separate locations in the Bsor area (Southern Israel). cv. Mose samples were collected in October 2014 from Ohad village. In May 2016, in Sde-Nitzan village, samples of cv. Odelia demonstrating unique fruit symptoms were collected (Fig 1I) compared to the typical symptomatic fruits of cv. Odelia (Fig 1G) and other commercial varieties (Fig 1F).

**Fig 4:**
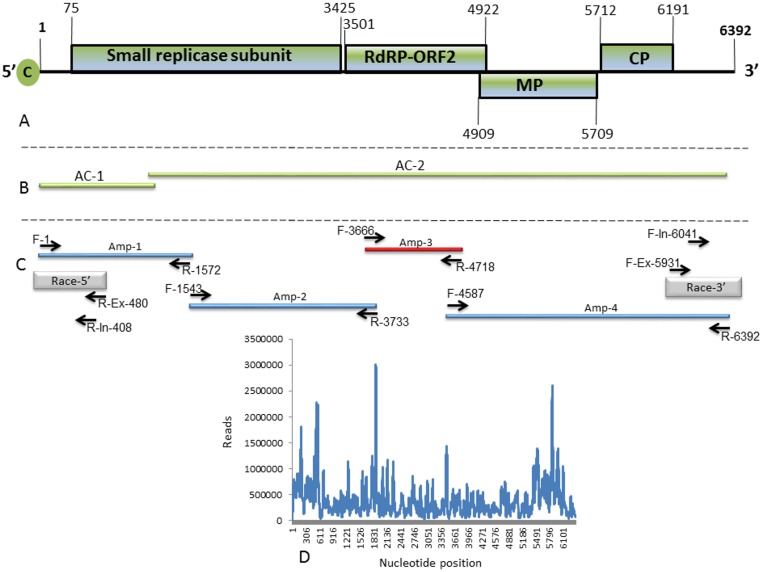
Schematic presentation of genome organization and sequencing strategy for retrieving the tobamovirus isolate genome sequence. (**A)** Schematic diagram of genome organization showing the viral four predicted ORFs. The numbers at the borders of each ORF represent the nucleotide base position of the start and termination codons of each ORF. (**B)** Illumina NGS analysis of samples from the outbreak at Ohad village. Lines represent the Illumina NGS assembled contigs (AC-1 and AC-2), obtained via whole-genome assembly analysis using tobamoviruses as a reference and de novo analysis using Velvet [29]. (**C)** Selected reverse transcription amplification (RT)-PCR and primer sets used to map and validate the complete viral genome. Grey lines represent 5’ and 3’ RACE used to obtain both viral untranslated regions (UTRs). (D) The distribution of the obtained small RNA along the viral genome.

The raw data obtained by the NGS Illumina MiSeq analysis contained 3,074,594 and 629,309 reads respectively. After 3' adaptor removal, low quality read removal and length-range filtering (19 to 35 bp), a total of 2,594,213 and 148,765 reads respectively remained; 51,414 and 9,630 of these were viral. The reads were mapped on the TMV genome (accession number EF392659.1), yielding 46% and 28% coverage of the TMV viral genome respectively, excluding the 5' and 3' ends. The distribution of the sRNA along the assembled and validated genome of the new tobamovirus isolate (Fig 4, see below) is depicted in Fig 4D. We performed *de-novo* assembly of sRNA raw data resulting in two large assembled contigs: contig 1 (928 nt), and contig 2 (5,418 nt) demonstrating the highest nucleotide sequence identity (85% and 82%, respectively), and amino acid sequence identity (95% and 93%, respectively) to the TMV Ohio strain complete genome (accession number FR878069.1). Notably, even though there were significant differences in disease symptoms between the tomato cvs. Mose and Odelia (Fig 1F and 1I), 100% nt sequence identity was obtained between the two NGS data sets of whole genome, with no sequence indication of other potential viruses. When the recent Jordanian tomato tobamovirus isolate sequence, (GenBank accession no. KT383474.1), was compared with our obtained sequence data we found 99% nt sequence identity and 99% aa sequence identity between the Israeli tobamovirus isolate and the Jordanian virus TBRFV-Jo.

### RT-PCR amplification for sequence validation and diagnostics

The sequence authenticity of the NGS data was verified by RT-PCR amplification using virion RNA as the template with sequence-specific primers. Sequence alignment allowed the design of primers according to the NGS reads and the assembled contigs of the new Israeli tobamovirus isolate (Fig 4B and 4C). In parallel, the 5’ and 3’ UTR’s obtained using RACE technique allowed the design of additional primer sets for RT-PCR amplification to validate the NGS-derived sequence and the entire genome of the virus. Four primer sets were generated in order to obtain the nucleotide sequences by RT-PCR amplicons. The primer locations in relation to the genome regions were at positions: 1 to 1572 (amplicon 1, Amp-1); 1543 to 3733 (Amp-2); 3666 to 4718 (Amp-3) and 4587 to 6392 (Amp-4) (Fig 4C and S5 Fig). The obtained amplicons followed by Sanger sequencing validated the authenticity of the NGS-derived sequence demonstrating 100% sequence identity. A primer set was designed to amplify tobamoviruses infecting tomatoes. A forward F-3,666 and reverse complement R-4,718 primers (Amp-3) (Fig 4C and S5 Fig) were designed based on a conserved ORF2 region observed between the new Israeli isolate and the *Solanaceae*-infecting tobamoviruses TMV, ToMV, ToMMV and the Jordanian new tobamovirus TBRFV-Jo deposited in the GenBank (accession number KT383474) (S5 Fig). This primer set was used successfully in diagnostic assays of tomato plants infected with the virus followed by Sanger amplicon sequencing for sequence verification (S5 Fig).

### Genome organization of the new Israeli isolate

**Fig 5:**
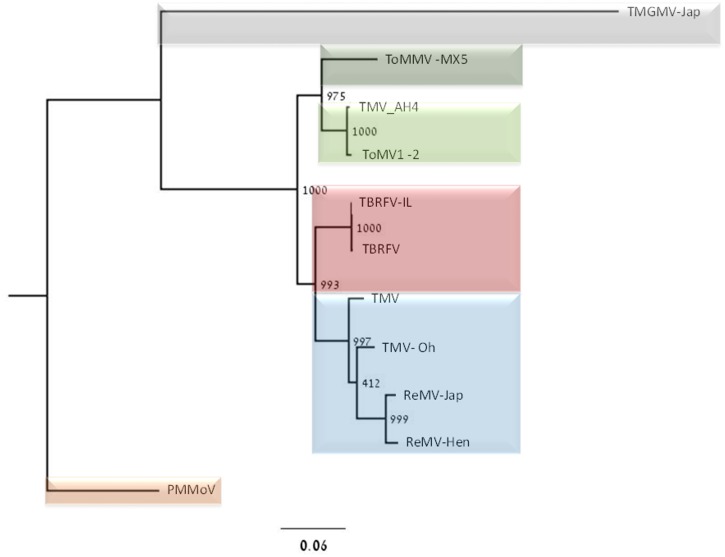
Rooted phylogenetic tree derived from the deduced amino acid sequences of the concatenated genes. The Israeli isolate of tomato brown rugose fruit virus (TBRFV-IL; KT721735); the Jordanian isolate of TBRFV (KT383474) and of several other viruses belonging to the genus *Tobamovirus*: *Tomato mottle mosaic virus* isolate MX5 (ToMMV-MX5; KF477193), *Tomato mosaic virus* isolate 1–2 (ToMV1-2; DQ873692), *Tobacco mosaic virus* (TMV; X68110), *Tobacco mosaic virus* isolate AH4 (TMV-AH4; KU321698), *Tobacco mosaic virus* isolate Oh (TMV-Oh; FR878069), *Rehmannia mosaic virus* isolate Jap (ReMV-Jap; AB628188), *Rehmannia mosaic virus* isolate Hen (ReMV-Hen; EF375551) and two outgroups: *Pepper mild mottle virus* (PMMoV; KX063611) and *Tobacco mild green mosaic virus* isolate Jap (TMGMV-Jap; AB078435). Each polyprotein-encoding sequence was aligned using the MAFT software for sequence alignments [26]. The tree was constructed based on maximum likelihood using the PhyML3.0 software with 1,000 bootstrap replicates [27].

The complete genome sequence of the new Israeli isolate comprised of 6,392 nucleotides was submitted to GenBank (accession no. KX619418). This genome sequence includes putative four ORFs of 1117, 474, 267 and 160 amino acids and two untranslated regions (UTRs) at the 5’ and 3’ ends of the viral genome (Fig 4A and 4C). The 5’ UTR includes 74 nucleotides. ORF1 starts with an AUG initiation codon located at positions 75 to 77 and terminates at a UGA stop codon located at positions 3423 to 3425. ORF2 starts with an AUG initiation codon located at position 3501 to 3503 and terminates at a UGA stop codon located at position 4920–4922. ORF3 starts with an AUG initiation codon located at positions 4909 to 4911 and terminates at a UGA stop codon located at positions 5707 to 5709. ORF4 starts with an AUG initiation codon located at positions 5712 to 5714 and terminates at a UGA stop codon located at positions 6189 to 6191. The 3’ UTR includes 201 nucleotides (Fig 4A and 4C). The predicted molecular weight of the sequenced CP using DNAMAN software is 17.497 kDa. The differences in nucleotide sequence between the Israeli strain and TBRFV-Jo were of four nucleotides with only a single substitution at position 3026 from T to C that was nonsynonymous resulting in a change of tyrosine (Y) to histidine (H) at position 986 of the aa sequence. The substitution is at ORF-1-2 encoding the RNA-dependent RNA polymerase (RdRp). The other three-nucleotide substitutions were at position 2533 from T to A, at position 3670 from G to A and at position 5607 from G to A. Phylogenetic tree analysis based on whole genome sequence shows that the new Israeli isolate, clustered with the Jordanian TBRFV-Jo virus, shares an ancestor with TMV clade (Fig 5).

### Epidemiology of the disease

**Fig 6:**
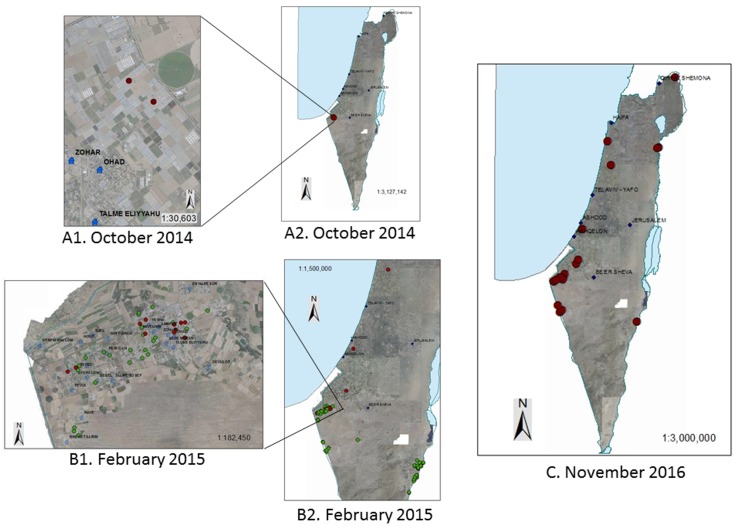
Monitoring the distribution of the new tobamovirus disease in tomatoes grown in greenhouses in Israel. **A1-A2**, The outbreak incident of viral infection in greenhouses of Ohad village in September-October 2014. **A1** Detailed picture of the infected area and surroundings. **A2**, The isolated occurrence of the disease depicted in Israel's map. **B1-B2**, Tomato disease spread as detected by the official Israeli PPIS survey on February 2015. **B1**, Detailed picture of the infected areas and surroundings. **B2**, Enlarged picture of the surroundings. **C**, The up to date disease status across the country in November 2016. Red dots represent positive detection of the virus tomato plants in the infected growing area. Blue dots represent negative detection of the virus in tomato plants.

The outbreak of the new tomato tobamovirus disease was an isolated event that was not treated, while detection of healthy tomato plants was received from various areas in the country. According to official inspectors of 'The agricultural extension services of Israel' there were no reports of disease symptoms prior to the September-October outbreak. However, in February 2015, in the four months following the outbreak, the disease spread to new tomato growing areas in the South of Israel (Melilot, Beit Ezra and Achituv), probably through the practice of visiting agronomists and professional inspectors or by transfer of non-tested contaminated seeds or seedlings. An official survey conducted by the Israeli PPIS started in February 2015 showed the isolated occurrence of the disease in the Bsor area in Southern Israel. While the remote areas of Ramat Negev and the Arava valley were detected negative for the virus (Fig 6A and 6B). At this time point, the tomato cultivars that were found infected with the new virus were cv’s: Mose and Ikram, non-grafted or grafted on Arnold or 'Beaufort' rootstocks, which represent the majority of the tomato cultivars that were grown at this time period in the infected area.

After 7 months, the disease spread to the Ramat Negev region, where growers specialize in the tomato cherry varieties (Fig 1D). Later on, the disease spread to the Arava valley in the Southeast and to the Beit Shean area in Northeast Israel. Since then the disease has become established nationally, in most of the protected structure grown tomatoes (Fig 6C). Additional cultivars harboring the ToMV resistance: Shiran, Diagrama-F1, 870, Whitney, Antonela, Magnolia became infected by the disease caused by the new tobamovirus isolate.

## Discussion

Tomato plants are grown worldwide in open fields, which often expose the plants to insect pests and vectors of plant viruses [30–32]. Growing trellised tomato plants inside protected structures (glasshouses, greenhouses and net-houses) has allowed control of entry of virus-transmitting insects. On the other hand, cultivation in protected structures exposes the plants to mechanically transmitted tobamoviruses due to intensive agro-techniques [6, 33, 34]. For decades, cultivating tomatoes in protected structures was achieved via the genotypes of the elite tomato varieties harboring the resistance genes *Tm-1*, *Tm-2* and *Tm-2*^*2*^ [8]. *Tm-2*^*2*^ was introduced by introgression from *L*. *peruvianum* to *L*. *esculentum* [8]. Although the tobamoviruses evolve rapidly, it was assumed that the changes in the viral MP that are necessary to break *Tm-2*^*2*^ resistance would reduce viral virulence [35]. Here we described an outbreak of a new tobamovirus disease in *Tm-2*^*2*^ resistant tomato plants, which occurred from October to November 2014 at Ohad village in Southern Israel. An outbreak of tobamiviruses requires immediate and full response at a very early stage, which includes quarantine of the contaminated area to prevent the disease from spreading. Successful eradication of new pathogen is challenged with limited success, although it may postpone disease establishment [36, 37]. Unfortunately, our current national experience, in which no eradication program has been adopted, resulted in disease incidences across the country. Currently the disease is established in most of the protected structures grown tomatoes in Israel (Fig 6C) and we are now developing a national disease management program.

It is quite clear now that the durability of the *Tm-2*^*2*^ resistance-conferring allele against ToMV in tomato plants has been jeopardized by the newly discovered tobamovirus isolate. Interestingly, the observed length range of the virus particles is wide, reminiscent of ToMV-R [38] and *Hibiscus latent fort pierce virus* (HLFPV) [39]. While small particles may represent breakdown products of the virus, the large particles over ~300 nm in length do not look like the product of mechanically accumulated aggregates (Fig 2A and S3 Fig).

ToMMV that causes tissue necrosis on leaves of tomato seedlings and mosaic and leaf distortion of mature plants has been reported in Brazil [40] and Mexico [18]. According to the published complete genome sequence [18], ToMMV is most closely related (85% identity) to ToMV. Phylogenetically ToMMV is clustered with the subclade of ToMV, TMV and *Rehmannia mosaic virus* (ReMV)[18]. The latter infects tomato as well and does not overcome the resistance genes [41, 42]. Following those reports, ToMMV was identified in U.S.A. [43–45], on pepper in China [46] and on tomatoes in Israel [47] and in Spain [48]. In contrast to the report of ToMMV on tomatoes in Israel, our epidemiological studies showed no evidence for ToMMV. We base our report on >1,000 ELISA tobamovirus-positive samples collected from most of the commercial tomato growers across the country, during the last two years (November 2014 -November 2016). Of these samples, 270 representative samples from all farms analyzed by RT-PCR followed by amplicon sequencing (Amp-3) revealed a single viral disease causing the current outbreak. An incident of double infection by the new Israeli tobamovirus isolate and TSWV has occurred showing severe symptoms (Fig 1H). Conclusively, we deduce that there is no infection by ToMMV in the areas investigated in the current study. We may therefore assume that the report regarding ToMMV infection of tomato crops in Israel reflects an isolated occurrence in a greenhouse of seed production-company. If indeed this is the case, a dire result might occur in which double infection of ToMMV and the currently studied isolate will spread. Recombination events between tobamovirus strains have been reported [49, 50]. In this potential scenario of double tobamovirus infection (the new tobamovirus isolate described in this current study and ToMMV), unknown consequences might occur regarding host range and symptom severity. As mentioned above, the 'Odelia' infection showed conspicuously different symptoms than those seen on cv. 'Mose' and on other samples collected in our survey (Fig 1F, 1G and 1I). In a single occurrence, the symptoms developed on cv. 'Odelia' were a late appearance of severe symptoms showing brown rugose pattern on the fruit, accompanied by dry necrotic symptoms on calyces and leaf mosaic and yellowing (Fig 1I). These late symptoms appeared only once, in a defined area in a tomato growing structure, while most of the plants exhibited the typical disease symptoms. Based on the small RNA-NGS analysis of the unique symptomatic 'Odelia' cultivar from Sde-Nitzan we found that there was no additional virus in the sample. It is possible that mixed infections with other pathogens have occurred. However, virus sequence of the Israeli isolate showed 99% sequence identity to the TBRFV-Jo (accession no. KT383474.1) recently published from Jordan. [19]. This new tobamovirus caused mild symptoms on the leaves with strong brown rugose symptoms on fruits of *S*. *lycopersicum* cv. Candela, which resemble the unique symptoms described above for the 'Odelia' variety. Phylogenetic analysis showed that the Jordanian isolate originated from a branch leading to TMV clade [19]. The high sequence identity between the Israeli isolate and the Jordanian tomato brown rugose fruit virus as well as the phylogenetic analysis (Fig 5) confirms that they originated from the same ancestral tobamovirus. Noticeably, the putative name suggested for the new tobamovirus discovered in Jordan does not reflect the disease symptoms caused by the virus discovered in Israel but only of unique symptoms discovered at one incident with cv. 'Odelia'. We have examined the possibility that the apparent brown rugose symptoms depend on susceptibility of the tomato variety 'Odelia' to the virus, which may occur under unique field conditions. However, the severe symptoms are not always apparent in infected 'Odelia' strain grown in various locations.

Regarding other members of the *Solanaceae* family, under certain circumstances the Israeli isolate is capable to infect pepper plants harboring the *L*^*1*,*3*,*4*^ resistance genes (Fig 3). Pepper plants are at a risk when planted on contaminated soil from previous growth cycle of infected tomato plants, especially in hot temperatures above 30°C, since the HR response on the root-stem often leads to plant collapse (Fig 3E–3G and S4 Fig). Interestingly, petunia plants are symptomless hosts, while eggplant and potatoes are non-hosts for the virus (Table 2). We are currently investigating the possibility of using grafted tomato plants on eggplant rootstock that may contribute to reduction of the primary inoculum when planting seedlings in contaminated soil.

## Conclusions

The current study identified a new tobamovirus isolate in Israel that infects tomato varieties harboring the *Tm-2*^*2*^ resistance grown in protected structures. The Israeli isolate is identical to the recently published tomato-infecting virus from Jordan. This virus and the lately worldwide-distributed ToMMV are a major threat for tomato crops. The Israeli isolate has unique symptoms on tomato plants and is capable of infecting *L*^*1*,*3*,*4*^ resistant pepper plants when cultivated on contaminated soil from previous growing cycle in high temperatures above 30°C. Common weeds, often asymptomatic when infected by the virus comprise a cryptic reservoir between growth cycles.

## Supporting Information

S1 Fig Inoculation of susceptible *Nicotiana tabacum* cultivars with tested tomato plant extracts (bioassay). Local lesions developed on *tabacum* cultivars following sap-mechanical inoculation of infected tomato plant extracts on (A). *N*. *tabacum* cv. Rustica. **(B).** *N*. *tabacum* cv. Samsun.
(TIF)

S2 Fig Non-infected tomato plants in field. (TIF)

S3 Fig Electron micrograph illustration of viral particles observed in leaf-dip preparations. (A-B) Distribution of viral particles lengths as imaged by TEM showing variability in particle sizes. Arrows indicating larger than 300 nm long particles.
(TIF)

S4 Fig Pepper plants harboring *L*^*1*,*3*,*4*^ hypersensitivity response (HR) to infection by the new tobamovirus isolate. **(D)** Necrotic lesions followed by dried apoptotic leaves. **(E)** HR symptoms developed following root inoculation demonstrating dried spots on stems leading to plant growth inhibition.
(TIF)

S5 Fig Alignment of the nucleotide sequences encoding the RdRp (ORF2) of five tobamovirus selected species. Line 1: *Tomato mosaic virus* (ToMV1-2; DQ873692); line 2: *Tomato mottle mosaic virus* (MX5; KF477193); line 3: Tomato brown rugose fruit virus (TBRFV-Jo; KT383474); line 4: *Tobacco mosaic virus* (TMV; X68110); line 5: Israeli isolate of tomato brown rugose fruit virus (TBRFV-IL; KX619418). Arrows represent the borders of the conserved regions, which served as a template for RT-PCR amplification. A designed general tobamovirus primer set: F-3666(TobGen) and R-4718(TobGen), encompassing the variable nucleotide sequences was used for species identification followed by amplicon sequencing using Sanger analysis.
(TIF)

S1 Table Primer sets for next generation sequencing (NGS) validation. (DOCX)
