# Supplementary figures and images for: A New Israeli Tobamovirus Isolate Infects Tomato Plants Harboring Tm-22 Resistance Genes
Source: PLoS One. 2017 Jan 20;12(1):e0170429. doi: 10.1371/journal.pone.0170429 (PMC5249172; doi:10.1371/journal.pone.0170429)

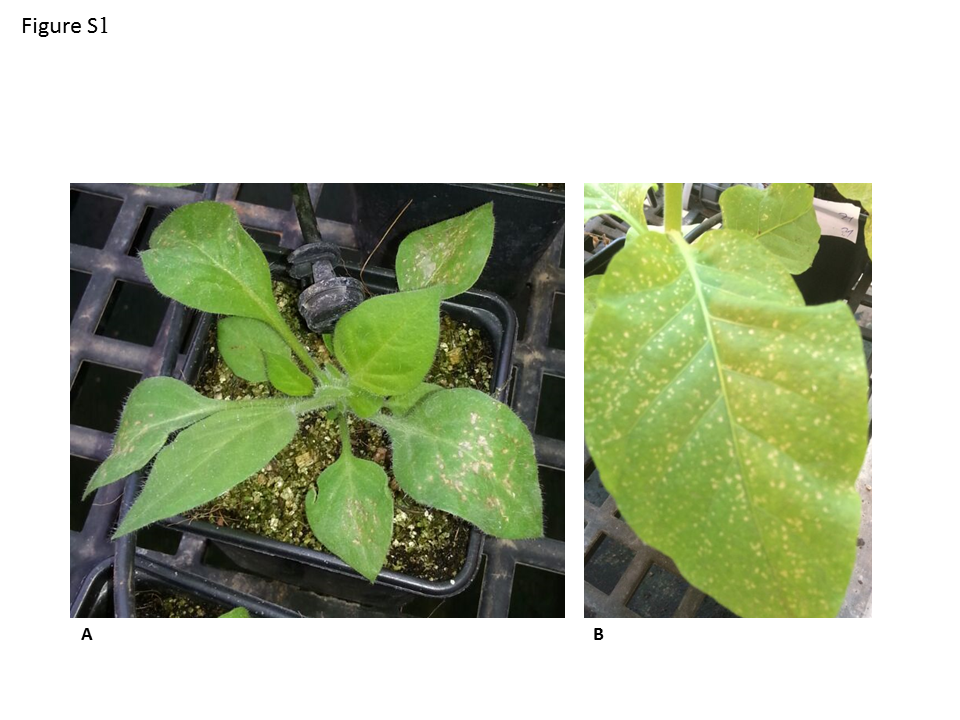

Supplement: S1 Fig — Local lesions developed on tabacum cultivars following sap-mechanical inoculation of infected tomato plant extracts on (A). N. tabacum cv. Rustica. (B). N. tabacum cv. Samsun. (TIF) [file pone.0170429.s001.TIF]

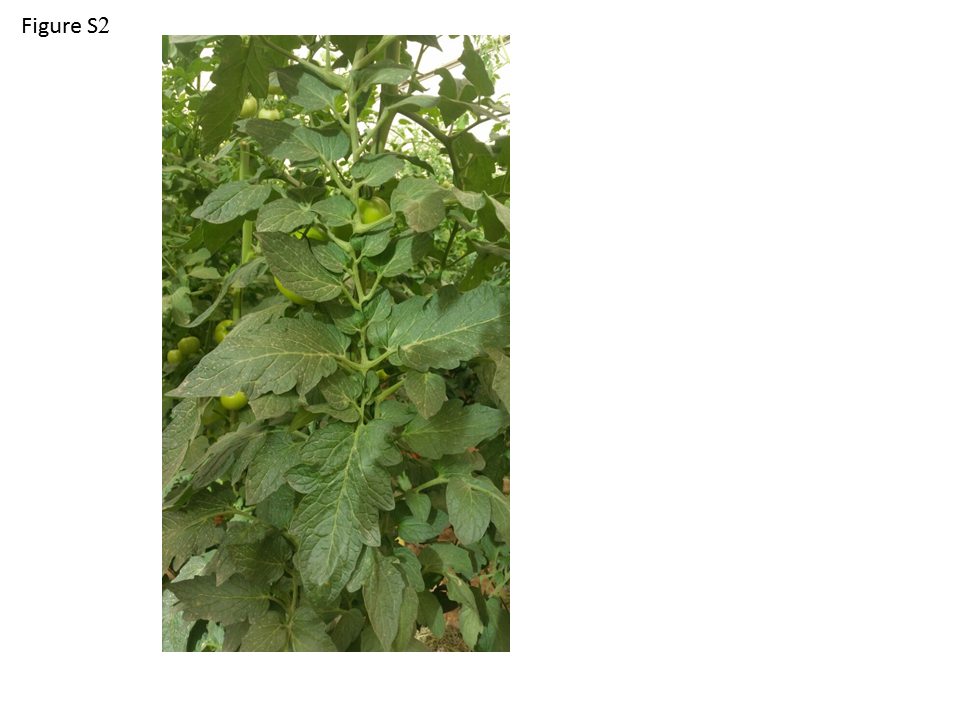

Supplement: S2 Fig — (TIF) [file pone.0170429.s002.TIF]

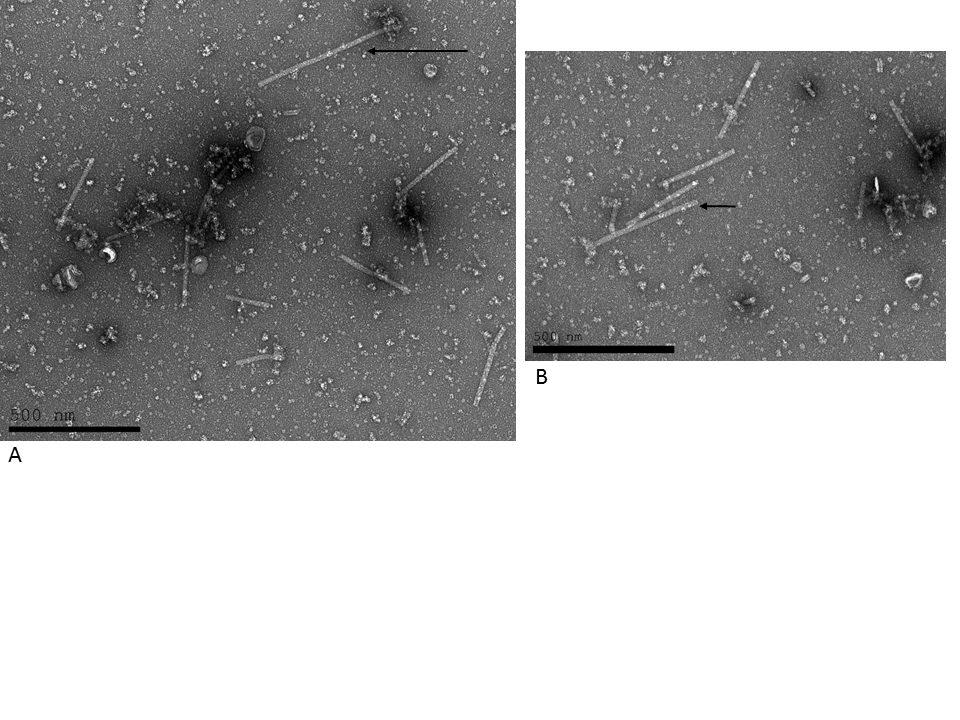

Supplement: S3 Fig — (A-B) Distribution of viral particles lengths as imaged by TEM showing variability in particle sizes. Arrows indicating larger than 300 nm long particles. (TIF) [file pone.0170429.s003.tif]

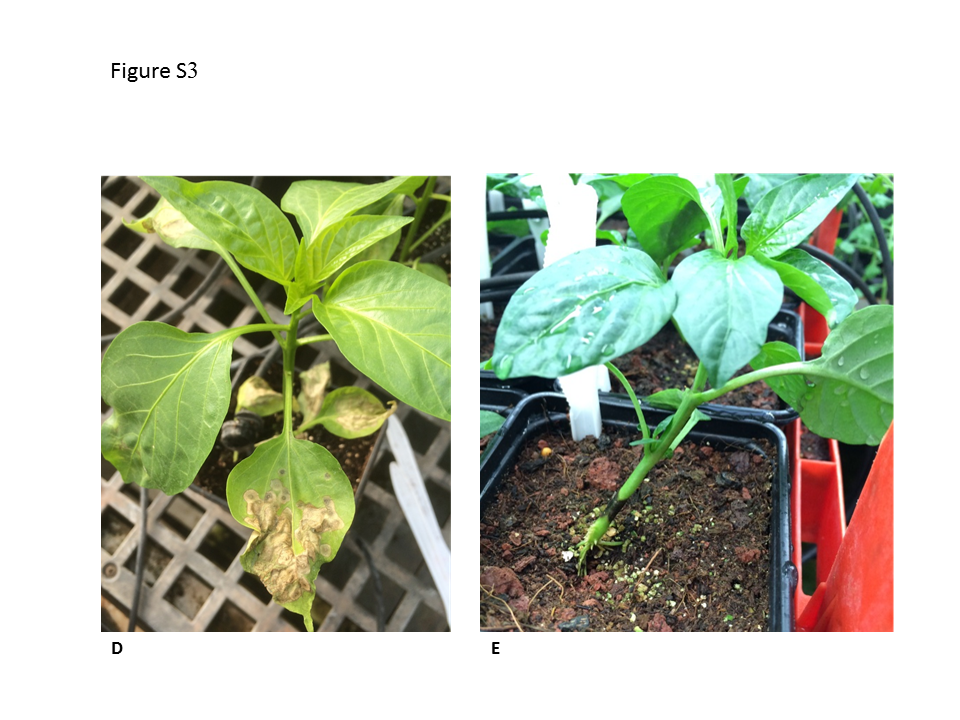

Supplement: S4 Fig — (D) Necrotic lesions followed by dried apoptotic leaves. (E) HR symptoms developed following root inoculation demonstrating dried spots on stems leading to plant growth inhibition. (TIF) [file pone.0170429.s004.TIF]

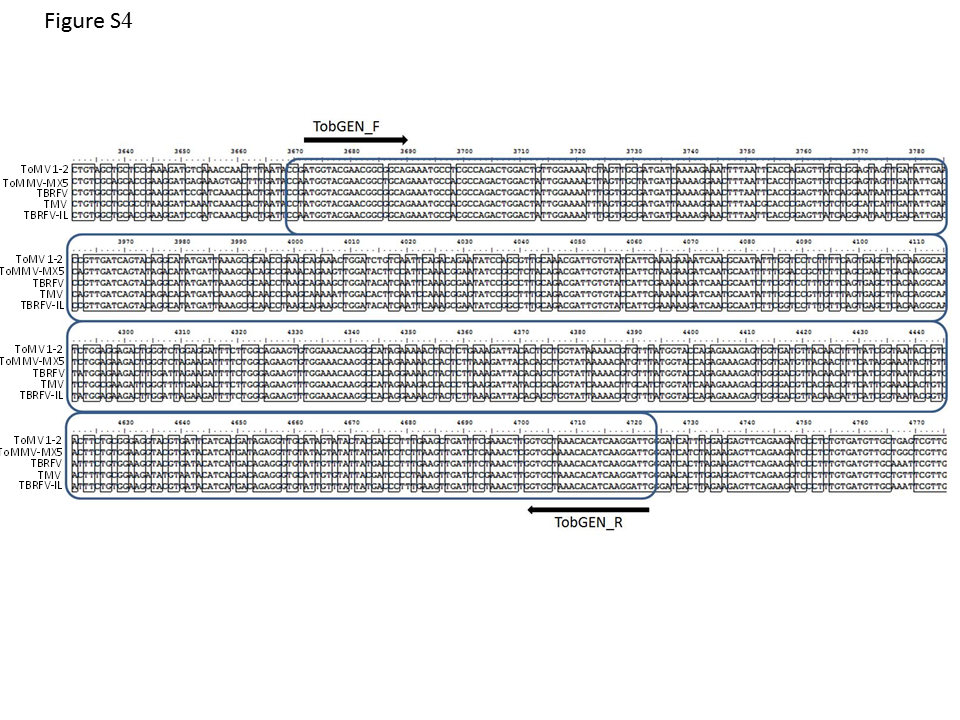

Supplement: S5 Fig — Line 1: Tomato mosaic virus (ToMV1-2; DQ873692); line 2: Tomato mottle mosaic virus (MX5; KF477193); line 3: Tomato brown rugose fruit virus (TBRFV-Jo; KT383474); line 4: Tobacco mosaic virus (TMV; X68110); line 5: Israeli isolate of tomato brown rugose fruit virus (TBRFV-IL; KX619418). Arrows represent the borders of the conserved regions, which served as a template for RT-PCR amplification. A designed general tobamovirus primer set: F-3666(TobGen) and R-4718(TobGen), encompassing the variable nucleotide sequences was used for species identification followed by amplicon sequencing using Sanger analysis. (TIF) [file pone.0170429.s005.TIF]
