# Supplementary material for: A New Israeli Tobamovirus Isolate Infects Tomato Plants Harboring Tm-22 Resistance Genes
Source: PLoS One. 2017 Jan 20;12(1):e0170429. doi: 10.1371/journal.pone.0170429 (PMC5249172; doi:10.1371/journal.pone.0170429)
Supplement: S1 Table — (DOCX) [file pone.0170429.s006.docx]

**S1 Table. Primer sets for next generation sequencing (NGS) validation.**

| **Sequence (5'----3')** | **Name (nt position)** | **Orientation** | **Primer  No.** |
| --- | --- | --- | --- |
| ATGGTACGAACGGCGGCAG | F-TobGEN (3666) | F | 1 |
| CAATCCTTGATGTGTTTAGCAC | R-TobGEN (4718) | C | 2 |
| GTGTATTTTTTACAACATATACCAAC | F-1(1) | F | 3 |
| CTAATGCGTCTCCCGACACT | R-1572 (1572) | C | 4 |
| AGATTTCCCTGGCTTTTGGA | F-1534 (1534) | F | 5 |
| ATCATCGCCACCAAATTTTC | R-3733 (3733) | C | 6 |
| CCAACCTTATGTGGAATTTCG | F-4587 (4587) | F | 7 |
| GAACCCCCGGTAGGGGCCCA | R-6392 (6392) | C | 8 |
| TTATGTCGCGGACATCAAGA | R-Ex-480 (480) | C | 9 |
| GTGAACATATGCCCGTCCTT | R-In-408 (408) | C | 10 |
| ACAATGCGGTACTAGATCCTCT | F-Ex-5931 (5931) | F | 11 |
| GACGCAACGGTGGCTATAAG | F- In -6041 (6041) | F | 12 |
